# Evaluation of a web-based, tailored intervention to encourage help-seeking for lung cancer symptoms: a randomised controlled trial

**DOI:** 10.1177/2055207620922381

**Published:** 2020-05-04

**Authors:** Julia Mueller, Alan Davies, Caroline Jay, Simon Harper, Chris Todd

**Affiliations:** 1 School of Health Sciences, University of Manchester, UK; 2 School of Computer Science, University of Manchester, UK; 3 Manchester Academic Health Science Centre, UK; 4 Manchester University NHS Foundation Trust, UK

**Keywords:** Health psychology, lung cancer, theory of planned behaviour, help-seeking, information-seeking, symptom appraisal

## Abstract

**Background:**

People with lung cancer often wait for several months before presenting symptoms to health services. Some patients report seeking information online to help them appraise symptoms. No research has evaluated whether websites about lung cancer present information in an optimal manner to encourage help-seeking.

**Objective:**

To evaluate the effectiveness of an online, tailored, theory-based intervention in encouraging help-seeking behaviour among people with potential lung cancer symptoms.

**Methods:**

The intervention consisted of a specialised website which provided tailored information about lung cancer and included a component to address beliefs about help-seeking, based on the Theory of Planned Behaviour (TPB-component). Individuals with undiagnosed symptoms were randomised to receive information about lung cancer in a factorial design (tailored/untailored × TPB-component/no TPB-component). Pre and post viewing webpages, participants reported perceived likelihood of seeking help. Data were analysed using robust mixed factorial ANOVA.

**Results:**

Data from 253 participants (73.9% female) were analysed. No effect for the TPB-component was found (*p* = 0.16), nor for tailoring (*p* = 0.27). Self-reported likelihood of seeking help increased significantly from pre to post (*p* < 0.001), regardless of tailoring and TPB-components.

**Conclusion:**

Self-reported likelihood of seeking help for potential lung cancer symptoms may increase after viewing information online. This does not appear to be affected by information tailoring and components to address beliefs. However, intentions remained unchanged in the majority of the sample. This suggests further efforts are needed to improve lung cancer websites if they are to be a useful resource for those seeking advice about their symptoms.

## Introduction

Lung cancer accounts for approximately 21% of all cancer deaths and 40,000 deaths per year in the UK.^1^ Tumours grow rapidly and are commonly detected at a late stage with metastatic spread.^2^ If lung cancer can be diagnosed earlier, one-year survival rates could be improved considerably, from 15–19% at Stage IV to 81–85% at Stage I.^3^ In the UK, low survival rates have been partly attributed to a larger proportion of late stage diagnoses,^4^ suggesting earlier diagnosis may improve survival rates.

The time to diagnosis is influenced by a multitude of factors, involving healthcare provider and system factors such as healthcare policies and resources, disease factors such as tumour growth rate, and patient factors such as cultural or psychological barriers to seeking help.^5–7^ Lung cancer patients often experience symptoms for several months before presenting to primary care.^8,9^ Reasons for delayed presentation to health services include lack of awareness of symptoms, attribution of symptoms to ageing or minor health conditions, masking of symptoms through co-morbid conditions and negative beliefs about help-seeking, such as fatalistic beliefs about the treatability of lung cancer.^8,10–15^

In the UK, several public health campaigns have endeavoured to raise awareness about lung cancer and encourage earlier presentation.^16–18^ These campaigns broadcast and circulate brief, simple messages mostly addressing the cough symptom, and have been associated with increases in knowledge levels, urgent general practitioner (GP) referrals for suspected lung cancer and lung cancers diagnosed.^17,18^ However, due to the strong emphasis on the cough symptom, awareness of other symptoms may remain low.^13,19^

Over the past two decades, the web has become an increasingly important health information source,^20^ often used to appraise undiagnosed symptoms.^21^ In a study with recently diagnosed lung cancer patients, 20% reported that they or someone among their next-of-kin had researched their condition online prior to diagnosis, and that their online research influenced their help-seeking behaviour.^22^ Thus, the web has the potential to play an important role in the time from perception of the first symptoms to presentation to health services and subsequently diagnosis. Many websites provide information about lung cancer symptoms and are possibly accessed frequently by those trying to decide whether they need to seek medical advice. However, no study to date has evaluated whether websites present information in an optimal way to encourage those with relevant symptoms to seek help.

The time from first detecting bodily changes to consulting a healthcare professional has been theorised to include two intervals:^23^ (a) the appraisal interval, which involves appraisal of symptoms, and (b) the help-seeking interval, which involves deciding whether to consult a healthcare professional. People with lung cancer experience barriers in both of these intervals. For example, people with lung cancer often do not appraise symptoms as requiring medical advice^13,24,25^ and, even if symptoms are perceived as serious, perceived negative consequences of help-seeking often prevent consultation with a healthcare professional.^8,10,12,24^ Previous research highlighted that people with lung cancer seek advice from health websites in both intervals.^22^

In this study, ‘help-seeking’ denotes the process from first perceiving symptoms to presenting (or not presenting) to healthcare services.^26^ Research indicates that help-seeking for lung cancer symptoms is often influenced by beliefs.^8,12,24,27^ For example, people with lung cancer report delaying help-seeking because they hold fatalistic beliefs about the treatability of lung cancer, because they fear blame and stigma due to smoking,^10,24^ or because of perceived difficulties due to limited availability of appointments.^25^ These findings suggest beliefs about the outcomes of help-seeking, normative beliefs about its acceptability and beliefs around the perceived level of control of the behaviour may play a role. These three belief types are captured in the Theory of Planned Behaviour (TPB).^28^ The TPB postulates that changing these beliefs will change intention to perform the behaviour, and ultimately the behaviour itself. Thus, incorporation of the TPB into the content of websites about lung cancer by addressing these key beliefs may enhance impact on help-seeking, but this has not been evaluated systematically.

Although previous interventions aiming to increase help-seeking for lung cancer symptoms have shown some impacts,^17^ effects appear to be limited due to low perceived personal relevance.^13,19^ A key advantage of web-based interventions is that they can be tailored to unique user characteristics using computing technology. Research indicates this increases perceived personal relevance, thereby producing stronger impacts on behaviour,^29^ including help-seeking for cancer symptoms.^30^

Thus, adapting websites about lung cancer in order to target beliefs about help-seeking (in accordance with the TPB) and tailoring information to individual users may increase the impact on help-seeking. The aim of this study is to test whether a tailored, TPB-based website about lung cancer can increase anticipated help-seeking among people with potential lung cancer symptoms.

Specifically, the tested hypotheses are:

**H1:** People with potential lung cancer symptoms who view online information about lung cancer with added TPB components to address beliefs about help-seeking, will show a larger increase in self-reported likelihood of visiting a doctor (comparing before to after viewing the information) than those who view the same information without TPB components.

**H2:** People with potential lung cancer symptoms who receive tailored information about lung cancer will show a larger increase in self-reported likelihood of visiting a doctor than those who receive untailored information.

Based on extant literature, it was not clear whether we should expect a combination of these two factors to produce stronger impacts on behaviour than each factor alone. Therefore, we also tested the following research question:

**RQ1:** Is there an interaction effect between tailoring and presence of TPB-components, such that those who receive both factors will show a different change in self-reported likelihood of visiting a doctor than those who receive only one factor, or none?

## Methods

### Study website

**Figure 1.**
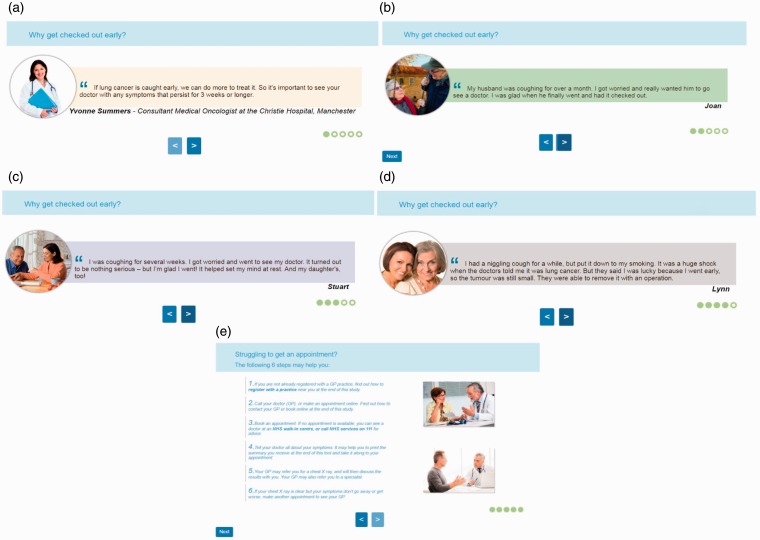
TPB-component presented to users. Sections (A–D) addressed behavioural beliefs by emphasising benefits of early help-seeking and the possibility of even mild symptoms being related to lung cancer. (A–D) also addressed normative beliefs by showing clinicians/family members who endorsed help-seeking. Section (E) targeted control beliefs.

To test our hypotheses, we developed a specialised website about lung cancer which incorporated tailoring and addition of components to address beliefs, based on the TPB. Our choice of these two factors was based on extant literature as outlined above, as well as extensive user engagement work.^27^ The website involves:

1. Tailoring: users first input information about their symptoms and risk factors into the website and are then presented with tailored information about lung cancer to aid with appraisal of the symptoms. For example, a younger user (<40 years) who reports being an ex-smoker and coughing up blood would subsequently be presented with detailed information on haemoptysis, as well as information about the prevalence of lung cancer among younger people, and among former smokers. The user would also be provided with tailored advice on whether an urgent referral for a chest X-ray may be indicated based on their age, symptoms and smoking status according to clinical guidelines for suspected cancer referral.^31^
2. Targeting beliefs about help-seeking via TPB-components: users are presented with a set of quotes by health professionals and (fictional) family members/friends to address beliefs about the outcomes and social acceptance of help-seeking, and a list of steps to secure an appointment with a GP, to enhance control beliefs. The set of quotations and the list of steps are referred to as the ‘TPB-component’ (Figure 1).

The website involves four pages which users clicked through consecutively: (a) page eliciting symptoms, age and smoking status; (b) page providing information about the reported symptoms and risk factors; (c) page displaying the TPB-component; and (d) page providing a summary (list of reported symptoms and risk factors) and tailored information regarding an urgent chest X-ray. More detail about the website is published elsewhere.^27^

Note that our website targeted processes in the appraisal interval (through tailored information about symptoms) and the help-seeking interval (by addressing beliefs about help-seeking), although the intervention was not developed specifically based on the Model of Pathways to Treatment.^23^

### Study design

Participants were randomised to one of four study groups (SGs), each receiving a different version of the website, in a 2 × 2 randomised factorial design with the two factors ‘presence of TPB-component’ (yes/no) and ‘information tailoring’ (tailored/untailored) to test for differential effects of tailoring and the TPB-component. Participants in the intervention group (INT) received tailored information with the TPB-component; participants in SG-Tail received tailored information, but no TPB-component; participants in SG-TPB received the TPB-component, but information was not tailored; and in SG-none, information was neither tailored nor supplemented by the TPB-component (Table 1). In the untailored groups, participants received information about all lung cancer symptoms and risk factors and generic information about urgent chest x-rays, regardless of the symptoms and risk factors they reported.

**Table 1.** Study groups (SGs).

	Tailored information about lung cancer symptoms and risk factors	Untailored information about lung cancer symptoms and risk factors
TPB-component	INT	SG-TPB
No TPB-component	SG-Tail	SG-none

### Participants

We recruited adults aged ≥18 years who reported experiencing any of the following undiagnosed symptoms (assessed upon entry to the website): a cough; a long-standing cough that changes or gets worse; dyspnoea; discomfort in the chest, shoulder or back; haemoptysis; hoarseness; unexplained weight loss or unexplained loss of appetite; swelling of face and/or neck; persistent/recurring chest infections; fatigue; or finger clubbing.

Recruitment strategies needed to be broad to target a large audience in order to identify individuals with relevant, undiagnosed symptoms. This included advertising the study via mailing lists (e.g. staff of various organisations), social media, advertising pages like Gumtree, and various websites (e.g. Salford Citizen Scientist). We also used Google Ad Words, which meant that the study link was displayed at the top of Google search results when terms related to lung cancer symptoms (e.g. ‘persistent cough’) were entered. As the website was based on UK guidelines,^31^ we included only people who reported living in the UK.

Sample size calculations were informed by unpublished pilot data. Determining the expected effect size was difficult, as, to our knowledge, no similar intervention evaluation is published in the literature (i.e. a web-based intervention to promote help-seeking for undiagnosed symptoms). In our pilot trial, the primary outcome (self-reported intention to seek help) was measured on a seven-point scale, with a pooled standard deviation of 2.033. Our pilot trial found no effect of the intervention but following the trial considerable changes were made to improve the intervention, the study design and the primary outcome measure. Table 2 shows the number of participants needed for mean differences of varying magnitude, based on α = 0.05 and 80% power. The sample size per group was multiplied by (1 – *r*^2^) to adjust for baseline measurement, where *r* is the expected correlation between the baseline and post-treatment measure, using a medium correlation of 0.3 as a conservative estimate. Based on Table 2, we determined that a minimum of 54 participants per group (*n* = 216) should be recruited.

**Table 2.** Sample sizes required to detect different mean differences (based on pilot data; primary outcome measured on a seven-point scale with SD = 2.033).

Mean difference	*n* per group	Correction for baseline measurement^a^
1.75	43	39
1.5	59	54
1.25	84	76
1	131	119

### Measures

#### Primary outcome: self-reported likelihood of seeking help

Participants responded to the question ‘How likely do you think it is that you will see a doctor about your symptoms in the next three weeks?’ on a 10-point scale from 0 to 100%. This item was measured at baseline and after viewing the web pages.

#### Initial survey

The website presented participants with an initial survey to elicit information used for tailoring (see Supplemental Material): a list of symptoms (including duration and severity); age (below/above 40 years, cut-off based on UK guidelines^31^); smoking status (current/former/never-smoker); and whether they were using the website for themselves or on behalf of someone else. This survey also asked whether they had previously seen a doctor about these symptoms. People with lung cancer often present to healthcare multiple times before receiving a referral and diagnosis,^32^ and in such cases individuals may be particularly inclined to consult the Web regarding symptoms.^22^ Therefore we included participants who had already seen a doctor, but users were advised to use the website only if their symptoms were still undiagnosed.

#### Demographics

Age, sex (male/female), ethnicity (White, Mixed/multiple ethnic groups, Asian/Asian British, Black/African/Caribbean/Black British, Other ethnic group, Prefer not to say) and educational level (none, primary school, some secondary school/high school, GCSE or equivalent, further education (e.g. A-levels, BTEC, HND), undergraduate university degree, postgraduate university degree) were measured. Gender and ethnicity measures were adapted from the census survey^33^ and the measure for educational level was adapted from Schneider et al.^34^ Education level was assessed as an indicator for socioeconomic position.^35^

#### Google Analytics

Data on website usage were collected via Google Analytics, a web analytics service which tracks and reports website traffic. Data used for this study were the number of unique users who visited the site, geographic location based on IP address, and the proportion of users who left the page with no further interactions (such as clicking on a link/button on the page).

### Procedure

**Figure 2.**
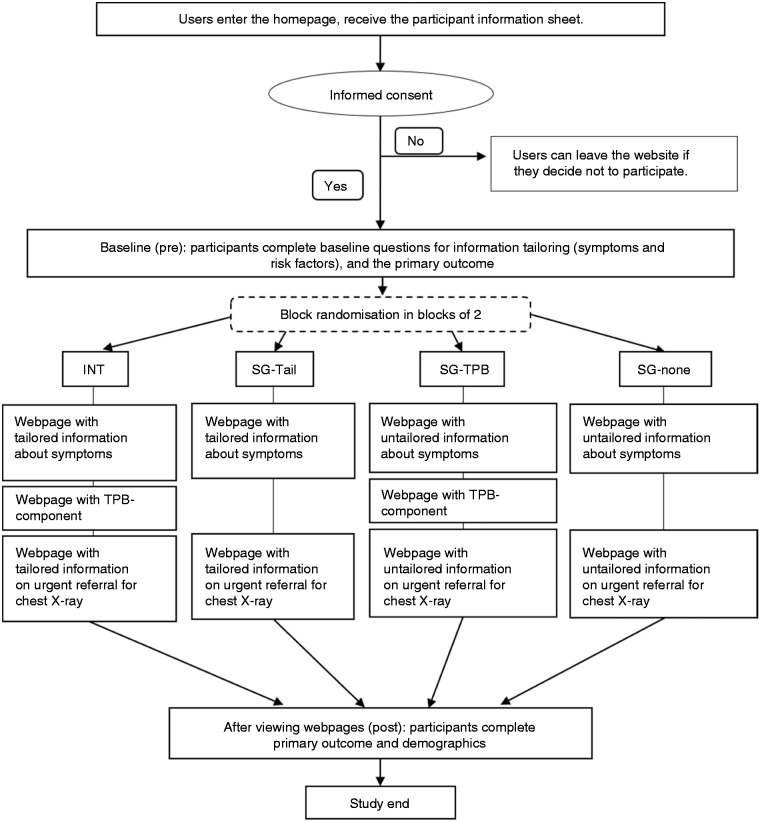
Study procedure. INT, intervention; SG, study group; TPB, Theory of Planned Behaviour.

The study took place online. On entry to the study website, participants were provided with the participant information sheet. After providing informed consent but before viewing any further webpages (baseline or ‘pre’ time point), all participants completed the initial survey as well as the primary outcome measure, self-reported likelihood of seeking help. Subsequently participants were block-randomised in blocks of two to one of the study groups, using the native randomisation function built into Python, the programming language used to develop the study website. Participants were exposed to information about lung cancer in different formats according to study group, as depicted in Figure 2. Webpages were shown to participants consecutively (participants clicked ‘next’ at the bottom of each page) rather than allowing participants to navigate freely across the website, to ensure that participants viewed all webpages. After viewing the webpages (‘post’ time point), participants completed the primary outcome measure again. Only participants who completed the primary outcome measure after viewing the webpages were included in the analysis, to ensure we included only those who viewed all pages.

### Statistical analyses

All statistical tests were carried out using a significance level of *α* = .05 and using either the statistical software package IBM SPSS Statistics 22 or the software environment R (Version 3.4.1.). The primary outcome measure was non-normally distributed. Therefore, to assess changes in self-reported likelihood of visiting a doctor from pre (baseline) to post (after viewing webpages) across the four study groups and to assess main and interaction effects of the two factors (tailoring and presence of the TPB component), we conducted a two-way mixed ANOVA using trimmed means as suggested by Wilcox as a robust method for data violating parametric assumptions,^36^ following consultation with several statisticians. For any significant effects, partial eta squared (η^2^) was computed as a standardised effect size measure, and assessed using the criteria 0.01, 0.06 and 0.14 for small, medium and large effects respectively.^37^ Partial eta squared was computed using the raw (untrimmed) data, in SPSS. To further provide an estimate of the magnitude of effects, the unstandardised trimmed mean difference is reported. The Kruskal–Wallis test was used to test for differences in means across the four study groups, and Dunn’s test was used to test pairwise comparisons post hoc where significant differences across groups were found. The Mann–Whitney *U*-test was used to test for differences between pairs of groups. To test for associations between categorical variables (e.g. study group and smoking status), we used Pearson’s chi-squared (*χ*^2^) test for independence or Fisher’s exact test where expected frequencies were below 5.

## Results

### Sample description

**Figure 3.**
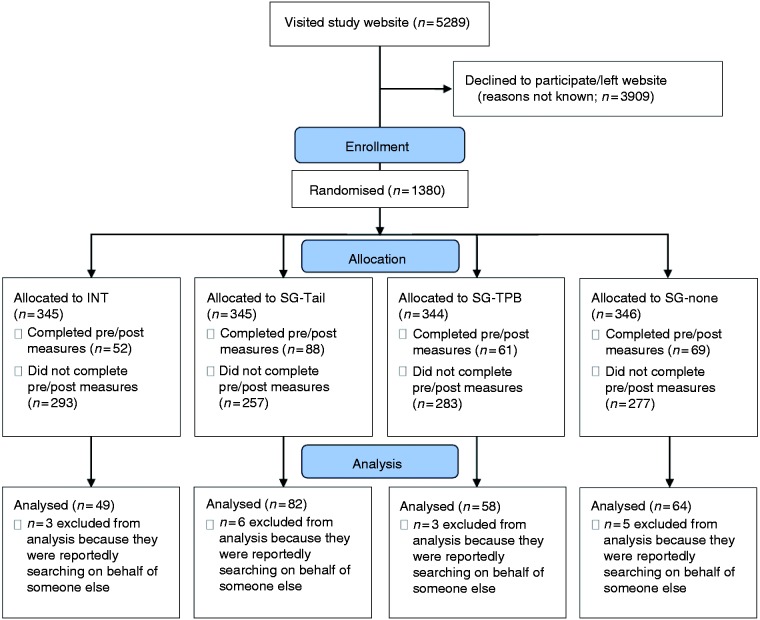
Flow diagram of the progress of participants through the study phases. INT, tailored information with TPB-component; SG-Tail, tailored information without TPB-component; SG-TPB, untailored information with TPB-component; SG-none, untailored information without TPB-component; TPB, Theory of Planned Behaviour.

Data were collected over a timeframe of four months. Figure 3 shows the progress of participants through the study phases. According to Google Analytics data, the majority of users had UK-based IP addresses (5101/5289, 96.4%). Overall, 23.1% (*c.* 1380 users) undertook further interactions on the website (such as clicking on the consent button, or on the ‘About’ page) after landing on the homepage, whereas 73.9% (*c.* 3909 users) left the homepage without further interactions. In total, 270 participants (19% of those who engaged with the website, 270/1380) completed the study (i.e. they viewed all webpages and completed the primary outcome). Seventeen participants indicated they were using the website on behalf of someone else and were excluded from analyses (*n* = 253).

Sample characteristics are shown in Table 3. Participants’ ages ranged from 18 to 86 years, (M = 43.1 ± 17.0, median = 44). The majority of participants were reportedly female (187/253, 73.9%) and of White ethnicity (222/253, 87.7%). Approximately half reported education levels below university degree level (135/253, 53.4%) and were smokers or ex-smokers (143/253, 56.5%). Despite using a block randomisation procedure, sample sizes across the four study groups differed considerably (Figure 3), indicating differential dropout.

**Table 3.** Self-reported sample characteristics.

Age (year)	
Range	18–86
Mean	43.1
SD	17.0
Median	44
	*n* (%)
Sex	
Female	185 (73.1)
Male	68 (26.9)
Education	
No education	6 (2.4)
Secondary school	17 (6.7)
Post-secondary school, e.g. GCSE	38 (15.0)
Further education, e.g. A-levels	74 (29.2)
Undergraduate degree	61 (24.1)
Post-graduate degree	57 (22.5)
Ethnicity	
White	222 (87.7)
Black	6 (2.4)
Asian	10 (4.0)
Mixed	6 (2.4)
Other	7 (2.8)
Prefer not to say	2 (0.8)
Smoking status	
Never smoker	110 (43.5)
Ex-smoker	77 (30.4)
Current smoker	66 (26.1)

a = *n* × (1 − *r*^2^) where *r* = 0.3

**Figure 4.**
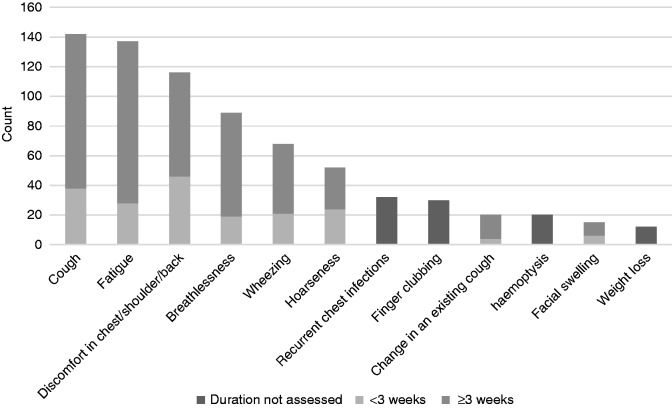
Frequency of symptoms across the sample (*n* = 253) and their respective duration. Where duration was not assessed, symptoms are considered warning signs regardless of duration.

As shown in Figure 4, the most commonly reported symptom was cough (142/253, 56.1%). Approximately a third of all participants (81/253, 32.0%) had already presented to health services with their symptoms prior to study participation.

### Group comparison at baseline and on sociodemographics

**Figure 5.**
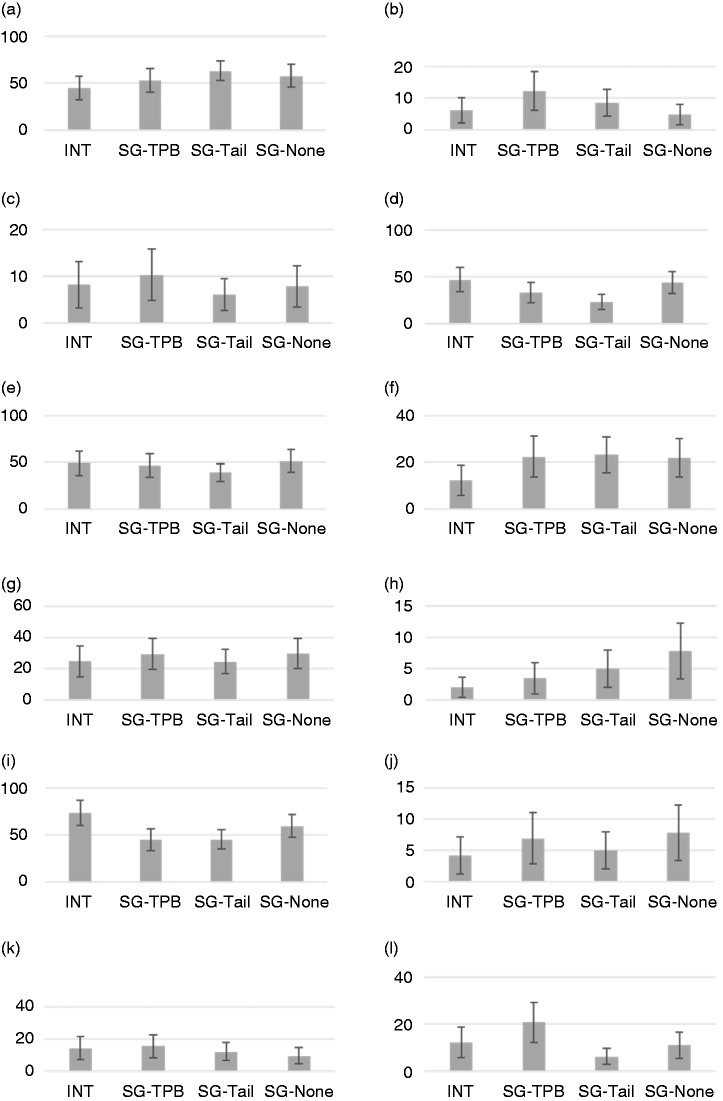
Percentage of participants reporting respective symptoms: (a) cough; (b) change in an existing cough; (c) haemoptysis; (d) breathlessness; (e) discomfort; (e) hoarseness; (g) wheezing; (h) unexplained weight loss; (i) fatigue; (j) swelling of face and/or neck; (k) persistent/recurring chest infections; (l) finger clubbing. INT, intervention group; SG-TPB, study group receiving TPB-component and generic information; SG-Tail, study group receiving tailored information but no TPB-component; SG-None, study group receiving generic information, no TPB-component. TPB, Theory of Planned Behaviour. Error bars are 95% confidence intervals.

The four study groups did not differ significantly in self-reported likelihood of seeking help at baseline (*χ*^2^ (3) = 0.45, *p* = 0.93). Groups differed significantly in reported age (*χ*^2^ (3) = 9.76, *p* = 0.02); participants in INT (M = 38.63, SD = 17.36) were significantly younger than participants in SG-Tail (M = 46.66, SD = 16.40) (*p* = 0.04). The proportion aged 40 or above in INT was 40.8% (20/49) compared to 69.5% (57/82) in SG-Tail. Chi-square tests did not indicate any significant differences across groups in terms of self-reported sex (*χ*^2^ (3) = 1.21, *p* = 0.75), smoking status (*χ*^2^ (3) = 5.09, *p* = 0.53), ethnicity (*χ^2^* (3) = 1.70, *p* = 0.64), or education level (*χ^2^* (3) = 2.49, *p* = 0.48). As indicated by 95% confidence intervals (CIs), the study groups did not differ in the proportion of participants reporting different symptoms (Figure 5(a)–(l)), excepting ‘finger clubbing’, where CIs for SG-TPB (12.3–29.2%) and SG-Tail (2.63–9.57%) did not overlap.

### Primary outcome (self-reported likelihood of visiting a doctor)

At baseline, participants across all groups reported a mean perceived likelihood of visiting a doctor of 43.0%, SD = 38.7, and after viewing webpages, participants scored on average 50.3%, SD = 38.70. Table 4 shows the change from pre to post across the four groups. The largest change in self-reported likelihood of visiting a doctor from pre to post was documented in INT (9.6%), whereas the smallest change was found in SG-none (3.9%). The Kruskal–Wallis test indicated no significant difference across the four study groups after viewing the webpages (*χ^2^* (3) = 4.98, *p* = 0.17).

**Table 4.** Mean self-reported likelihood of seeking help before and after intervention, and change from pre to post viewing webpages, across the four study groups.

Study group	*n*	Baseline self-reported likelihood of visiting a doctor (pre) (M (95% CI))	After viewing webpages self-reported likelihood of visiting a doctor (post) (M (95% CI))	Change (pre- to post) (M (95% CI))
INT (tailored, with TPB-component)	49	40.20 (29.20–51.21)	49.80 (38.18–61.41)	9.59 (5.05–14.13)
SG-Tail (tailored, without TPB-component)	82	43.29 34.93–51.65)	51.22 (42.92–59.52)	7.93 (3.38–12.48)
SG-TPB (untailored, with TPB-component)	58	46.38 (35.37–57.39)	54.48 (44.03–64.93)	8.10 (4.12–12.09)
SG-none (untailored, without TPB-component)	64	41.72 (32.35–51.09)	45.63 (36.13–55.12)	3.91 (–0.33–8.14)

Results from the two-way mixed ANOVA using trimmed means showed a significant change from pre to post, with participants reporting significantly higher likelihood of visiting a doctor after viewing the information (regardless of tailoring and presence of the TPB-component) as compared to before (*p* < 0.001), with a large effect size of η_p_^2^ = 0.15 (an increase of 11.8%). There was no significant effect of the TPB-component on the change from pre to post (*p* = 0.16), nor of tailoring (*p* = 0.27), thus not supporting Hypotheses 1 or 2. Regarding RQ1, we found no significant interaction effect between the TPB-component and tailoring on the change from pre to post (*p* = 0.66).

Overall, self-reported likelihood of seeking help increased in 30.8% of the sample (78/253), decreased in 4% (10/253) and remained the same in 65.2% (165/253). Those whose intention increased did not differ significantly from the remaining sample in terms of sex (*χ^2^* (1) = 0.00, *p* = 0.99), smoking (*χ^2^* (2) = 0.07, *p* = 0.97), education (*χ^2^* (5) = 9.08, *p* = 0.11), ethnicity (*χ^2^* (5) = 6.2, *p* = 0.29), age (*U* = 6069.5, *p* = 0.16), or whether they had already seen a doctor about their symptoms (*χ^2^* (1) = 2.11, *p* = 0.15). They also did not differ significantly in terms of the symptoms reported (see Supplemental Material).

## Discussion

This study aimed to determine whether novel forms of presenting information online (i.e. information tailored to users’ characteristics, and addition of components to target users’ beliefs around help-seeking) can increase individuals’ self-reported likelihood of seeking medical help for symptoms potentially related to lung cancer. We found no significant effects of either tailoring or TPB-components. Self-reported likelihood of visiting a doctor increased significantly after viewing the online information, but this did not depend on whether the information was tailored or supplemented by the TPB-component. This suggests the current mode of presenting information about lung cancer online – generic, untailored information without specific theory-based components – may be sufficient to enhance help-seeking behaviour. However, as we did not compare the website against a group with no intervention, it is unclear whether this increase resulted from viewing the information. It is also possible that simply eliciting participants’ symptoms during the initial survey increased intention to seek help.^9^ Self-reported likelihood of seeking help remained the same in the majority of the sample, indicating that further efforts are needed to improve websites about lung cancer to harness their potential in reducing delays to presentation.

The TPB-component did not significantly affect changes in perceived likelihood of seeking help, thus not confirming Hypothesis 1. TPB constructs can be difficult to change, and behavioural beliefs appear to be particularly resistant to change.^38^ Beliefs which may impede help-seeking such as worry about wasting the doctor’s time and fear of stigmatisation are likely deep-rooted and based on previous experiences,^24,39^ and may be difficult to change in a one-off intervention lasting only 15–20 minutes. Furthermore, it should be noted that the TPB postulates that *salient* beliefs influence intention. Eliciting salient, relevant beliefs in a given context requires careful consideration and wording.^40^ Our approach drew on previous research on reasons for delayed help-seeking in lung cancer^27^ and it is possible that not all salient beliefs were identified, as previous research has relied on retrospective reports of events leading up to diagnosis, which can be subject to bias.^41,42^

Another important consideration is that the TPB postulates that not only belief strength but also the value the individual places on this belief influence intention. For attitudes, we targeted both belief strength (e.g. emphasising that even mild symptoms can be related to lung cancer) and the associated value (emphasising favourable outcomes such as early detection). For subjective norms, we placed a stronger emphasis on enhancing belief strength. Motivation to comply (i.e. the value) was assumed because we selected social reference groups (clinicians and family members) who have been shown to act as facilitators to help-seeking.^10,43–45^ In terms of perceived behavioural control, we focused on the perceived power of certain factors to facilitate or impede help-seeking (i.e. the value) rather than addressing the perceived probability that these factors are present (i.e. belief strength). As such, there are certain aspects of the TPB that our intervention did not fully implement.

Moreover, there are a variety of different approaches to (health) behaviour change, including learning and cognitive theories, stage models, affect-based approaches and social cognition theories.^46^ The TPB was selected following a systematic and rigorous mapping process,^27^ however employing a different theoretical basis may lead to different outcomes.

Tailoring also appeared to have no effect on changes in participants’ self-reported likelihood of seeking help, thus not confirming Hypothesis 2. It is difficult to compare our tailoring approach to those used in other studies, as articles generally provide little detail on how tailoring was achieved. Tailoring is often treated as a unitary construct, with interventions merely described as ‘tailored’ or ‘untailored’, but tailoring spans a wide range of different strategies and methods.^47^ Further research is required to determine the optimum type and degree of tailoring for this context, and future studies should report their tailoring approach in detail.

Finally, it is possible that wording on our website reassured participants that their symptoms did not warrant help-seeking. However, all content was worded carefully during our preparatory work with clinicians and members of the public^27^ to avoid false reassurance.

### Strengths

Many studies exploring web use for symptom appraisal and its effect on help-seeking use hypothetical symptom scenarios with healthy participants.^21^ In contrast, this study recruited participants who (reportedly) experienced actual symptoms, thus increasing external validity. Due to its mixed factorial design, the trial allowed us to compare how participants’ perceptions of their likelihood of seeking help changed from before to after viewing information, and to assess whether the degree of change differed depending on how the information was presented. We were also able to assess the two factors (tailoring and presence of the TPB-component) separately as well as their interaction. Thus, the design provides more useful insights than a simple intervention/control comparison. Although the self-reported primary outcome measure is subject to reporting bias, it enabled the assessment of alternate forms of help-seeking such as accessing walk-in centres, which primary-care records may not include.

### Limitations

Sample sizes across the four study groups were uneven indicating differential dropout of participants. Differential dropout can bias study findings, particularly when it occurs for systematic rather than random reasons.^48^ Reasons for the differential dropout are unclear. SG-Tail retained the highest number of participants. This group was also the shortest (least number of pages). The two groups with the TPB-component retained the lowest number of participants, suggesting those who dropped out either took issue with this component itself, or with the extra page this entailed. The amount of text does not appear to have played a role: INT and SG-TPB had the same number of pages but SG-TPB involved more text, yet SG-TPB retained more participants than INT. Overall our findings suggest differential dropout may have occurred due to differing number of webpages. However, in our pilot study all groups had the same number of pages, and this did not mitigate the issue. We were unable to assess why users left the website, because browsers do not allow forced redirection on exit of a website. Prior to implementing the website, we had tested it with a small number (*n* = 5) of potential users in a Think Aloud evaluation to identify any issues. Perhaps further testing in a laboratory setting with a larger sample would have helped to shed light on this.

We found that participants in INT were significantly younger than participants in SG-Tail. This may have biased findings because younger and older adults tend to assess online health information differently.^49^ It should also be noted that information was tailored based on participants’ age and, according to NICE guidelines, participants aged above 40 with certain symptoms were advised that an urgent chest X-ray may be indicated. As SG-Tail had a higher proportion of participants aged over 40, more participants in this group received advice regarding an urgent chest X-ray. INT and SG-Tail were designed to be identical excepting the TPB-component, however, the differential effects of age mean that the tailored information differed between the two groups, thus potentially masking/attenuating effects of the TPB-component.

A larger proportion of participants in SG-TPB reported ‘finger clubbing’ than in SG-Tail. This could potentially bias findings as some symptoms prompt help-seeking more than others,^50^ though the literature does not presently indicate how finger clubbing would affect help-seeking. However, study groups did not differ in the proportions of participants reporting any of the remaining 11 symptoms, indicating the groups were largely similar in their symptom profiles.

Study findings may also be biased by the large proportion of female participants (73%). Women are more likely than men to seek health information online,^20^ and the difficulty of engaging men in health interventions is well documented in the literature.^51^ Our results may not accurately reflect men’s responses, and more needs to be done to engage men. Finally, it should be noted that the sample showed a higher proportion of young participants, participants with university-level education and non-smokers than would be expected from a typical lung cancer population,^52^ raising concerns about generalisability to the lung cancer population. Future endeavours to harness the web in encouraging earlier presentation should allocate sufficient resources to developing strategies to target at-risk groups.

It should also be noted that our study was powered to detect a medium effect size based on Cohen’s criteria,^37^ therefore smaller but significant effects may have been missed.

### Practice implications

Previous endeavours to increase help-seeking for lung cancer have mainly focused on the cough symptom, and on brief, simple messages, targeting the broad population.^17,18^ Findings from this study can complement these efforts by showing how online information can help inform those with relevant symptoms, taking the varied symptom profile of lung cancer into account. We found that self-reported likelihood of seeking help increased significantly after viewing online information about lung cancer, suggesting online resources could play a role in promoting earlier presentation. However, self-reported likelihood of seeking help did not change in the majority of the sample. This suggests further efforts are needed to improve websites about lung cancer if they are to be useful in terms of prompting those seeking advice about their symptoms to approach health services.

## Conclusion

The findings of this study suggest that presenting information about lung cancer online can potentially increase self-reported likelihood of seeking medical advice, though whether this information is tailored or supplemented with theory-based components did not appear to play a role. This potential should be further explored. Theory-based components to address beliefs about help-seeking did not appear to affect intention to seek help in this study, nor did tailoring information to individual users. However, various methods of information tailoring exist^29^ and there is a broad range of possible strategies to address TPB constructs.^38^ A different mode or degree of tailoring and different strategies to address health beliefs (or selection of a different theory) might produce different outcomes and thus further research should be devoted to web-based approaches. Research shows awareness of cancer symptoms is associated with cancer survival,^53^ and the web as a cost-effective and widely accessed health information source should be utilised in the endeavour to increase awareness. Future campaigns seeking to increase cancer awareness and early help-seeking through online resources need further research to inform evidence-based design.

## Supplemental Material

sj-pdf-1-dhj-10.1177_2055207620922381 - Supplemental material for Evaluation of a web-based, tailored intervention to encourage help-seeking for lung cancer symptoms: a randomised controlled trial Click here for additional data file. Supplemental material, sj-pdf-1-dhj-10.1177_2055207620922381 for Evaluation of a web-based, tailored intervention to encourage help-seeking for lung cancer symptoms: a randomised controlled trial by Julia Mueller, Alan Davies, Caroline Jay, Simon Harper and Chris Todd in Digital Health

sj-pdf-2-dhj-10.1177_2055207620922381 - Supplemental material for Evaluation of a web-based, tailored intervention to encourage help-seeking for lung cancer symptoms: a randomised controlled trial Click here for additional data file. Supplemental material, sj-pdf-2-dhj-10.1177_2055207620922381 for Evaluation of a web-based, tailored intervention to encourage help-seeking for lung cancer symptoms: a randomised controlled trial by Julia Mueller, Alan Davies, Caroline Jay, Simon Harper and Chris Todd in Digital Health
