# Supplementary material for: Evaluation of a web-based, tailored intervention to encourage help-seeking for lung cancer symptoms: a randomised controlled trial
Source: Digit Health. 2020 May 4;6:2055207620922381. doi: 10.1177/2055207620922381 (PMC7218332; doi:10.1177/2055207620922381)
Supplement: sj-pdf-2-dhj-10.1177_2055207620922381 - Supplemental material for Evaluation of a web-based, tailored intervention to encourage help-seeking for lung cancer symptoms: a randomised controlled trial [file sj-pdf-2-dhj-10.1177_2055207620922381.pdf]

Appendix A: Initial survey

What is your age?

- ○ Under 40
- ○ 40 and over

Do you smoke?

- ○ Yes, I currently smoke
- ○ No, but I used to smoke
- ○ No, I have never smoked

Which of the following symptoms do you have?

(Tick all that apply)

[Note that, upon selection of a symptom, additional questions appear to assess duration and intensity]

- ☐ A cough
- ☐ A change in a **long-standing** cough
- ☐ Coughing up phlegm with blood in it, or spitting blood
- ☐ Feeling breathless
- ☐ Discomfort in the chest, shoulders or back
- ☐ A change in the voice (such as hoarseness, sounding deeper, weaker, or quivering)
- ☐ Noises when breathing (such as wheezing or harsh breath sounds)
- ☐ Unintentional weight loss
- ☐ Feeling more tired than usual
- ☐ Any swellings or lumps around the face or neck area

☐ Recurring or persistent chest infections

☐ Changes in the appearance of fingers or fingernails

Have you already seen a doctor about your symptoms?

○ Yes

○ No

Are you researching your own symptoms, or on behalf of someone else?

○ Own symptoms

○ Someone else

Appendix B

Expected frequencies and observed frequencies of symptoms in participants whose self-reported intention to seek help increased from pre to post (n=78), compared to those where intention remained the same (n=165) or decreased (n=10).

		Intention decreased or stayed the same (n=175)<br>n (expected count)	Intention increased from pre to post (n=78)<br>n (expected count)	Chi-square χ^2(df)	*p (2-sided)*
Cough	Yes	100 (98.2)	42 (43.8)	χ^2 (1)=0.24	0.63
	No	75 (76.8)	36 (34.2)		
Change in existing cough	Yes	16 (13.8)	4 (6.2)	χ^2 (1)=1.19	0.27
	No	159 (161.2)	74 (71.8)		
Haemoptysis	Yes	15 (13.8)	5 (6.2)	χ^2 (1)=0.35	0.65
	No	160 (161.2)	73 (71.8)		
Breathlessness	Yes	61 (61.6)	28 (27.4)	χ^2 (1)=0.03	0.87
	No	114 (113.4)	50 (50.6)		
Discomfort in chest /shoulder/ back	Yes	82 (80.2)	34 (35.8)	χ^2 (1)=0.23	0.63
	No	93 (94.8)	44 (42.2)		
Hoarseness	Yes	33 (26.0)	19 (16.0)	χ^2 (1)=1.00	0.32
	No	142 (139.0)	59 (62.0)		
Wheezing	Yes	43(47.0)	25 (21.0)	χ^2 (1)=1.54	0.22
	No	132 (128.0)	53 (57.0)		
Weight	Yes	9 (8.3)	3 (3.7)	χ^2 (1)=0.20	0.76
	No	166 (166.7)	75 (74.3)		
Fatigue	Yes	97 (94.8)	40 (42.2)	χ^2 (1)=0.37	0.54
	No	78 (80.2)	38 (35.8)		

Facial swelling/lumps	Yes	9 (10.4)	6 (4.6)	χ^2 (1)=0.63	0.43
	No	166 (164.6)	72 (73.4)		
Recurring chest infection	Yes	19 (22.1)	13 (9.9)	χ^2 (1)=1.65	0.20
	No	156 (152.9)	65 (68.1)		
Finger clubbing	Yes	18 (20.8)	12 (9.2)	χ^2 (1)=1.34	0.25
	No	157 (154.2)	66 (68.8)		
